# I thought it was a hemangioma! A pictorial essay about common and uncommon liver hemangiomas’ mimickers

**DOI:** 10.1186/s13244-024-01745-1

**Published:** 2024-09-19

**Authors:** Matteo Bonatti, Riccardo Valletta, Valentina Corato, Tommaso Gorgatti, Andrea Posteraro, Vincenzo Vingiani, Fabio Lombardo, Giacomo Avesani, Andrea Mega, Giulia A. Zamboni

**Affiliations:** 1 Department of Radiology, Hospital of Bolzano (SABES-ASDAA), Teaching Hospital of Paracelsus Medical University (PMU), Bolzano, Italy; 2 Department of Radiology, IRCCS Ospedale Sacro Cuore - Don Calabria, Negrar (VR), Italy; 3 Department of Radiology, Radiation Oncology and Hematology, Fondazione Policlinico Universitario, A. Gemelli IRCCS, Rome, Italy; 4 Department of Gastroenterology, Hospital of Bolzano (SABES-ASDAA), Teaching Hospital of Paracelsus Medical University (PMU), Bolzano, Italy; 5 Department of Diagnostics and Public Health, Institute of Radiology, University of Verona, Policlinico GB Rossi, P.Le LA Scuro 10, 37134 Verona, Italy

**Keywords:** Liver neoplasms, Hemangioma, MRI, CT, Differential diagnosis

## Abstract

**Abstract:**

Focal liver lesions are frequently encountered during imaging studies, and hemangiomas represent the most common solid liver lesion. Liver hemangiomas usually show characteristic imaging features that enable characterization without the need for biopsy or follow-up. On the other hand, there are many benign and malignant liver lesions that may show one or more imaging features resembling hemangiomas that radiologists must be aware of. In this article we will review the typical imaging features of liver hemangiomas and will show a series of potential liver hemangiomas’ mimickers, giving radiologists some hints for improving differential diagnoses.

**Critical relevance statement:**

The knowledge of imaging features of potential liver hemangiomas mimickers is fundamental to avoid misinterpretation.

**Key Points:**

- Liver hemangiomas typically show imaging features that enable avoiding a biopsy.
- Many benign and malignant liver lesions show imaging features resembling hemangiomas.
- Radiologists must know the potentially misleading imaging features of hemangiomas’ mimickers.

**Graphical Abstract:**

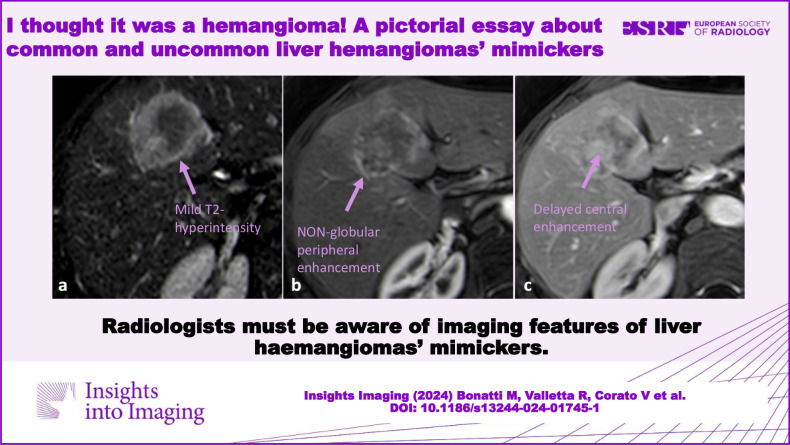

## Introduction

Liver hemangiomas represent the most common primary benign liver tumor, with a prevalence of 2–20% in autoptic studies and a female-to-male ratio of 2:1–5:1 [1–3]. Hemangiomas are increasingly encountered in everyday clinical practice because of the huge amount of performed imaging studies. According to published series, hemangiomas may be detected in 5% of CT examinations and in 2–4% of US examinations [4–6]. Hemangiomas have peculiar imaging features that enable accurate diagnosis using contrast-enhanced imaging modalities [2]. No percutaneous biopsy or surgical resection is needed in cases of lesions with typical US appearance in nononcological patients with “healthy liver”, as well as in lesions with a typical enhancement pattern [7]. On the other hand, many benign and malignant liver lesions may show misleading imaging features that radiologists must be aware of in order to avoid misdiagnosis [8].

In this article we will review multimodality imaging features of liver hemangiomas and show a series of potential hemangiomas mimickers (Table 1).

**Table 1:** Multimodality imaging features of liver hemangiomas and their mimickers

	US	CT	MRI	Contrast enhancement
**Cavernous hemangioma**	Homogeneously hyperechoic with acoustic enhancement	Hypodense	Hypointense on T1, markedly hyperintense on T2, no diffusion restriction (shine through artifact), hypointense on HBP	Peripheral globular arterial enhancement with progressive centripetal filling (vessels-like)
**Capillary hemangioma**	Usually homogeneously hyperechoic with acoustic enhancement	Iso/hypodense	Hypointense on T1, markedly hyperintense on T2, no diffusion restriction, hypointense on HBP	Rapid complete arterial enhancement without washout (vessels-like)
**Focal nodular hyperplasia**	Iso/slightly hypointense; spoke-wheel pattern at Doppler	Slightly hypodense	Isointense on T1, slightly hyperintense on T2, no diffusion restriction, isointense ± hyperintense rim on HBP	Rapid arterial enhancement (centrifugal) with subsequent iso-enhancement; on CT/MRI, central scar with delayed enhancement
**Focal Hepatic steatosis**	Hyperechoic, ill-defined, no acoustic enhancement	Hypodense	Signal drop on out-of-phase T1, isointense on T2, no lesion on DWI, isointense on HBP	Iso-enhancement
**Inflammatory hepatocellular adenoma**	Hyperechoic surrounded by hypoechoic halo	Variable	Iso/hyperintense on T1, heterogeneously hyperintense on T2, variable diffusion restriction, hypointense on HBP (20% iso/hyperintense)	Vivid arterial enhancement followed by iso-enhancement
**Arteriovenous fistula**	No solid lesion	No solid lesion	No solid lesion, possible focal flow void	Focal vessel-like enhancement
**Angiomyolipoma**	Heterogeneously hyperechoic	Heterogeneously hypodense with fat-density areas	Markedly hyperintense on T1 and T2 with hypointensity after fat saturation; hypointense on HBP	Arterial enhancement of the non-fat components; hypoenhancement of the fat components
**Intrahepatic cholangiocarcinoma**	Intermediate echogenicity surrounded by a hypoechoic halo	Hypodense	Heterogeneously hypointense on T1, heterogeneously hyperintense on T2, peripheral rim of increased diffusion restriction, hypointense on HBP (possible contrast retention!)	Peripheral rim of arterial enhancement with subsequent slow centripetal filling (No central filling on CEUS!)
**Mucinous colorectal metastases**	Hyperechoic	Markedly hypodense	Markedly hypointense on T1, markedly hyperintense on T2, no increased diffusion restriction (shine through artifact), hypointense on HBP (possible contrast retention!)	Peripheral rim of arterial enhancement with progressive central filling (No central filling on CEUS!)
**Angiosarcoma**	Heterogeneously hyperechoic	Heterogeneously hypodense	Hypointense on T1, heterogeneously hyperintense on T2, peripheral diffusion restriction, hypointense on HBP	Irregular foci of peripheral arterial enhancement with progressive incomplete filling
**Hemangioendothelioma**	Heterogeneously hypoechoic	Heterogeneously hypodense	Heterogeneously hypointense on T1, heterogeneously hyperintense on T2, heterogeneous diffusion restriction, hypointense on HBP	Variable, often peripheral arterial enhancement

*HBP* hepatobiliary phase

## Liver hemangiomas

### Histopathologic features

At pathology, liver hemangiomas appear as well-delineated blue-red lesions that tend to collapse on sectioning. They consist of blood-filled vascular spaces lined by a flattened endothelium and supported by fibrous septa. Three histological subtypes of liver hemangiomas have been described [9]. Cavernous hemangiomas are the most common histological subtype and may become very large [10]. They are prevalently constituted by large vascular spaces with minimal amounts of fibrous septa; thrombosis and calcification may be observed, typically in larger lesions. Capillary hemangiomas are usually small ( < 2 cm) and are characterized by small vascular spaces surrounded by extensive connective tissue. Sclerosed hemangiomas are the consequence of extensive fibrosis following thrombosis and obliteration of vascular spaces; they are also known as thrombosed or hyalinized hemangiomas [11].

### Typical imaging features

**Fig. 1:**
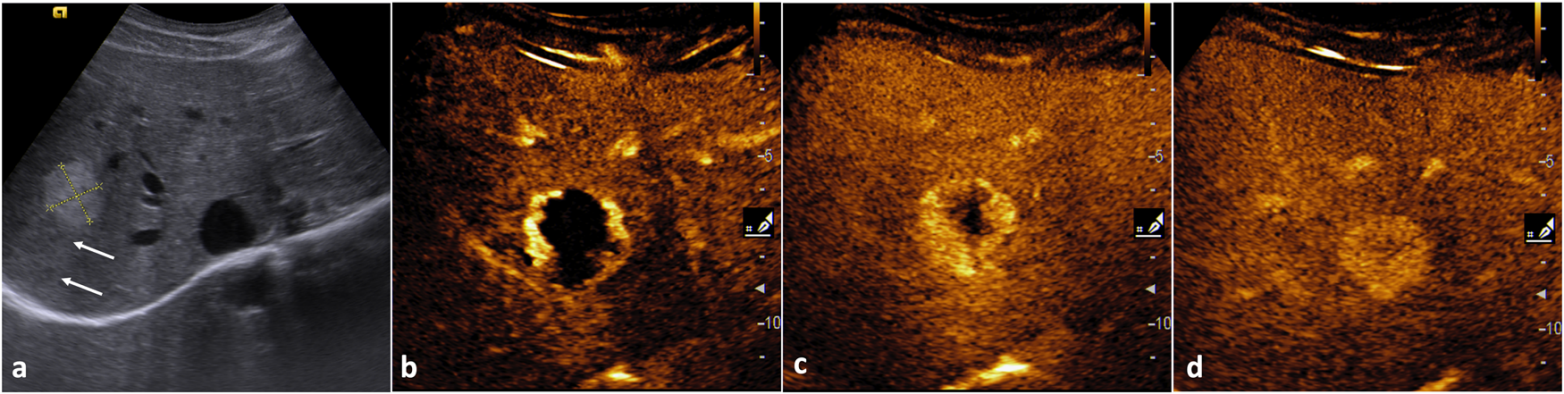
Typical US findings in cavernous hemangioma. On B-mode US (**a**) liver hemangioma appears as a well-defined homogeneously hyperechoic mass with posterior acoustic enhancement (arrows). Contrast-enhanced US (**b–d**) shows a globular centripetal progressive enhancement pattern

US often represents the first-line imaging modality for evaluating liver hemangiomas. In the US, hemangiomas usually appear as homogeneously hyperechoic solid masses with sharp margins and posterior acoustic enhancement (Fig. 1). This pattern is typical for cavernous hemangiomas because of the high number of interfaces between vascular spaces and fibrous septa and of the slow blood flows, but capillary and sclerosed hemangiomas may appear iso-hypoechoic [12, 13]. Large lesions usually show lobulated margins and tend to become inhomogeneous because of degenerative changes and thrombosis [14, 15]. The adjacent liver parenchyma shows normal echogenicity and no peritumoral halo should be observed.

Contrast-enhanced ultrasound (CEUS) represents an excellent problem-solving technique in newly US-detected liver hemangiomas. Cavernous hemangiomas typically show peripheral discontinuous globular enhancement during the arterial phase followed by progressive centripetal filling during the portal venous phase leading to iso-enhancement relative to adjacent vessels in the late phase; avascular lacunae may be present [16, 17] (Fig. 1). Capillary hemangiomas show rapid centripetal complete enhancement during the arterial phase. Atypical enhancement patterns may be observed, especially in lesions showing atypical features at the basal US [18].

**Fig. 2:**
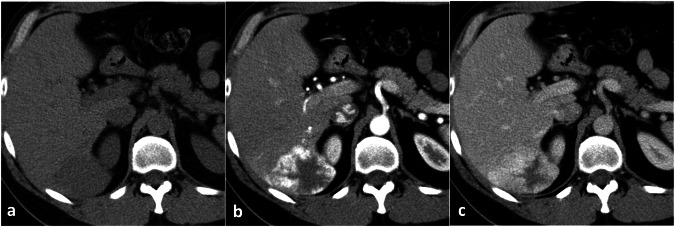
Typical CT findings in cavernous hemangioma. On unenhanced CT (**a**) the lesion appears slightly hypodense in comparison to liver parenchyma. After contrast material administration (**b**, **c**), an incomplete globular centripetal progressive enhancement pattern should be observed

On CT, liver hemangiomas typically appear hypodense compared to the adjacent parenchyma on unenhanced acquisitions showing density values similar to hilar vessels because of their vascular lacunae [14]. On post-contrast acquisitions, they typically show the same enhancement pattern described for CEUS, i.e., peripheral discontinuous globular arterial enhancement with progressive centripetal filling in cavernous hemangiomas (Fig. 2) and complete arterial filling in capillary hemangiomas [11]. The enhancing globules must show density values like the adjacent vessels [19]. No satellite nodules should be observed, whereas a transient hepatic attenuation difference (THAD) may surround small flash-filling hemangiomas, probably because of the presence of intralesional artero-portal shunts [20].

**Fig. 3:**
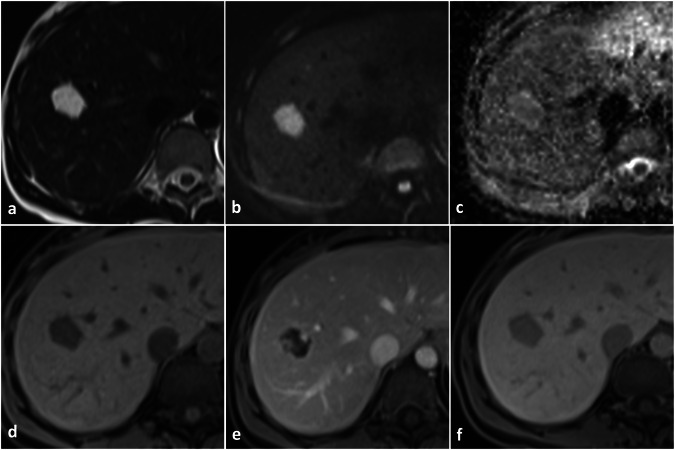
Typical MRI findings in cavernous hemangioma. Liver hemangioma appears markedly hyperintense on T2-weighted images (**a**) determining shine through artifact on high b value DWI (**b**) without true diffusion restriction on ADC map (**c**). The lesion appears hypointense on T1-weighted images (**d**), shows globular peripheral enhancement in the arterial phase (**e)** and appears hypointense in the hepatobiliary phase (**f**)

MRI has the highest accuracy in characterizing liver hemangiomas [14]. MRI protocol for focal liver lesions’ characterization must include at least in/opposite phase T1-weighted sequences, FSE T2-weighted sequences, diffusion-weighted sequences (high b value of at least 800 s/mm^2^) with ADC map, dynamic gradient echo T1-weighted sequences after liver-specific contrast material injection, including late arterial phase, portal venous phase and equilibrium phase (180 seconds after contrast injection), and hepatobiliary phase gradient echo T1-weighted sequences (10–30 minutes after contrast injection if using Gd-EOB-DTPA, 45–90 minutes after contrast injection if using Gd-BOPTA) [21]. On MRI, hemangiomas appear markedly hyperintense on T2-weighted images (the so-called “light bulb sign”) due to the fluid content of vascular lacunae [22, 23] and hypointense on T1-weighted images. Because of their low cellularity, hemangiomas do not show increased diffusion coefficient restriction on DWI, but shine-through artifacts on high b-value images are common because of the high signal intensity on T2-weighted images. The dynamic enhancement pattern is the same as described for CEUS and CT, i.e., peripheral discontinuous globular arterial enhancement with progressive centripetal filling in cavernous hemangiomas and complete arterial filling in capillary hemangiomas; pseudo-washout may be observed in the equilibrium phase after Gd-EOB-DTPA administration [14]. Hemangiomas appear hypointense in the hepatobiliary phase after hepatospecific contrast material administration due to the absence of hepatocytes, but pseudo-enhancement (i.e., lesion’s signal intensity slightly higher when comparing hepatobiliary phase to pre-contrast T1-weighted images) may be observed because of contrast material persistence within the dilated cavernous vessels [2, 3] (Fig. 3 and S1).

In non-oncologic patients with a “healthy liver”, US is enough for the diagnosis of characteristic lesions smaller than 3 cm. On the other hand, contrast-enhanced imaging (CEUS, CT, or MRI) is mandatory in US-atypical lesions and in oncologic patients or patients with underlying liver disease [3, 7]. Imaging follow-up is not indicated once the diagnosis is established [3].

### Atypical imaging features

Liver hemangiomas may show atypical imaging patterns that do not allow definitive radiological diagnosis and often lead to biopsy to rule out malignancy. The most common atypical hemangioma is the sclerosed subtype, which shows extensive fibrosis substituting vascular spaces [24] determining reduced signal intensity on T2-weighted images (Figure S2). On contrast-enhanced studies there is a loss of the typical peripheral globular enhancement. Moreover, massive fibrotic reaction may determine capsular retraction [25].

## Benign mimickers

### Focal nodular hyperplasia

#### Histopathologic features

Focal nodular hyperplasia (FNH) is the second most common benign solid liver lesion, and it is also often incidentally detected during imaging studies [8]. According to the WHO, FNH should not be considered a benign neoplasm, but a hyperplastic response of hepatocytes to a pre-existing vascular anomaly [26]. A prominent central feeding artery is usually observed, surrounded by a fibrotic radial fibrous scar and by hypertrophic hepatocytes. The biliary drainage system is often malformed and insufficient.

#### Typical imaging features

Given its histological composition, FNH has echogenicity, density, and signal intensity like adjacent normal liver. At the US, FNH is often barely recognizable, appearing slightly hypoechoic compared to the adjacent liver. The so-called spoke-wheel pattern may be observed on color-Doppler [27]. On unenhanced CT, FNH is usually slightly hypodense, whereas, on unenhanced MRI, it is often recognizable only on T2-weighted images as a slightly hyperintense lesion. On contrast-enhanced studies, FNH shows hyperenhancement in the arterial phase with a relatively hypo-enhancing central scar, followed by substantial iso-enhancement in the following phases of the dynamic study with central scar relative hyperenhancement. Thanks to its real-time evaluation, CEUS may enable the highlighting of the centrifugal arterial filling of the lesion [28]. On the other hand, MRI with the use of hepatospecific contrast material allows for confirmation of the presence of functioning hepatocytes in the lesion that appear isointense in the hepatobiliary phase with possible hyperintense peripheral rim due to insufficient bile outflow [29–31].

#### Misleading imaging features

**Fig. 4:**
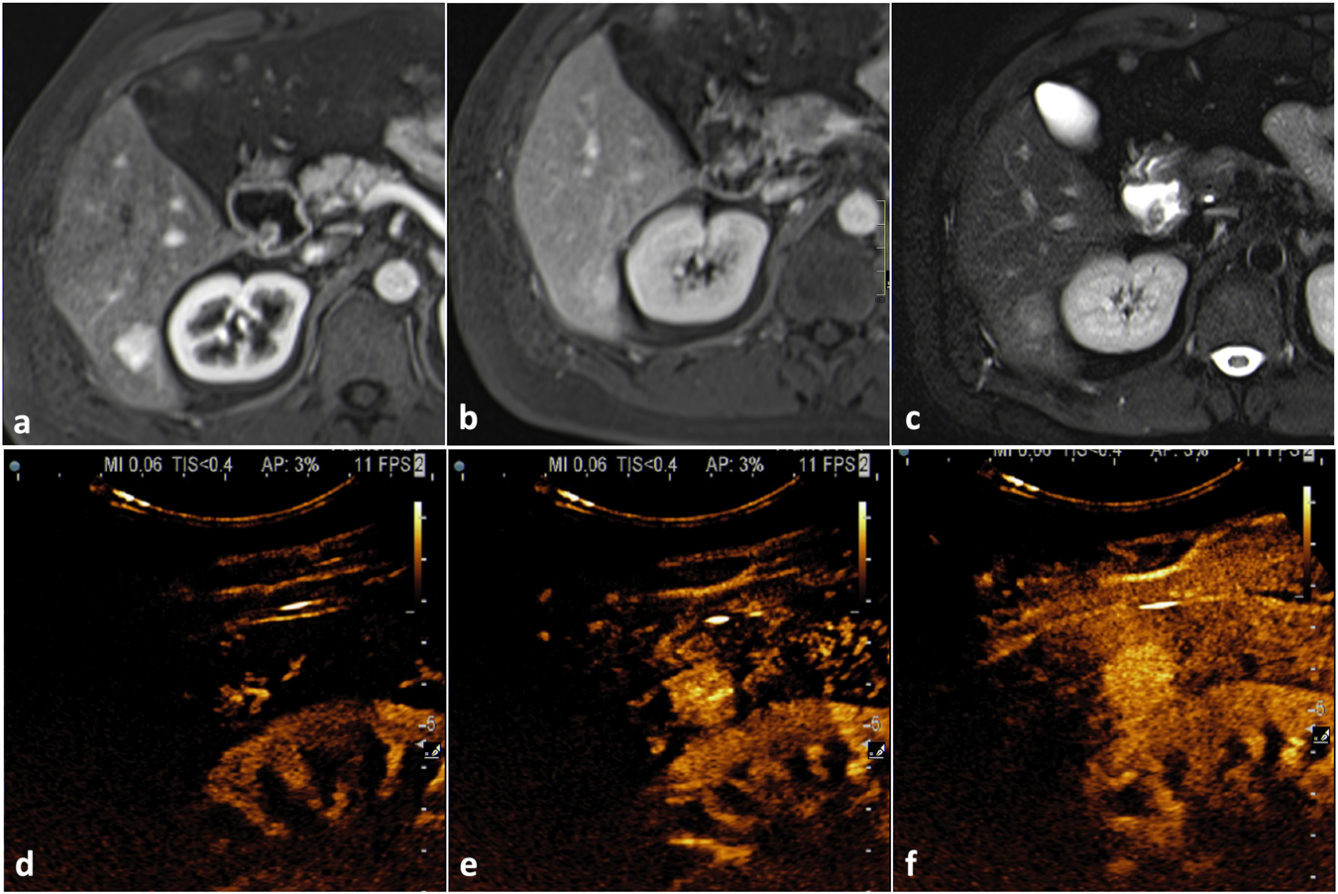
Focal nodular hyperplasia (FNH). The lesion shows strong arterial enhancement (**a**), but its signal intensity is lower than adjacent vessels in the portal venous phase (**b**). Moreover, the lesion appears only mildly hyperintense on T2-weighted images (**c**). On contrast-enhanced US the lesion appears homogeneously hypervascular in the late arterial phase (**f** ), but rapid centripetal filling can be depicted in earlier arterial phases (**d**, **e**)

On contrast-enhanced CT and MRI, a solid lesion showing arterial phase hyperenhancement with no wash-out may be misinterpreted as liver hemangioma (Fig. 4); this is particularly true for small FNHs that may resemble capillary hemangiomas.

#### Tricks to avoid misinterpretation

FNHs’ enhancement has no globular pattern; in lesions larger than 2 cm, this feature enables to safely exclude the diagnosis of a hemangioma. Moreover, FNHs appear hypointense in comparison to adjacent vessels in portal venous and equilibrium phases, and isointensity is appreciable on hepatobiliary phase MR images. Substantial iso-intensity/density on unenhanced imaging and the absence of a “light bulb sign” on T2-weighted images enable one to exclude the diagnosis of hemangioma in lesions of every size. In the US, FNHs show no hyperechogenicity, and centripetal enhancement may be depicted on CEUS.

### Focal hepatic steatosis

#### Histopathologic features

Focal steatosis refers to an area of increased intrahepatocyte fat content within the liver parenchyma [32, 33]. The most probable pathophysiologic mechanism underlying focal steatosis is the presence of an aberrant vein communicating with intrahepatic portal branches, leading to a focal perfusion change with subsequent metabolic alterations [34].

#### Typical imaging features

Focal steatosis appears as an ill-defined area of increased echogenicity in the US, usually in a subcapsular location. On unenhanced CT, focal steatosis shows slight hypodensity compared to a healthy liver. Signal intensity drop is observed on out-of-phase phase T1-weighted images, whereas the lesion appears isointense to the adjacent liver on all other MRI sequences [35, 36]. In contrast-enhanced imaging studies, focal steatosis shows the same enhancement dynamic of the adjacent liver in every phase.

#### Misleading imaging features

**Fig. 5:**
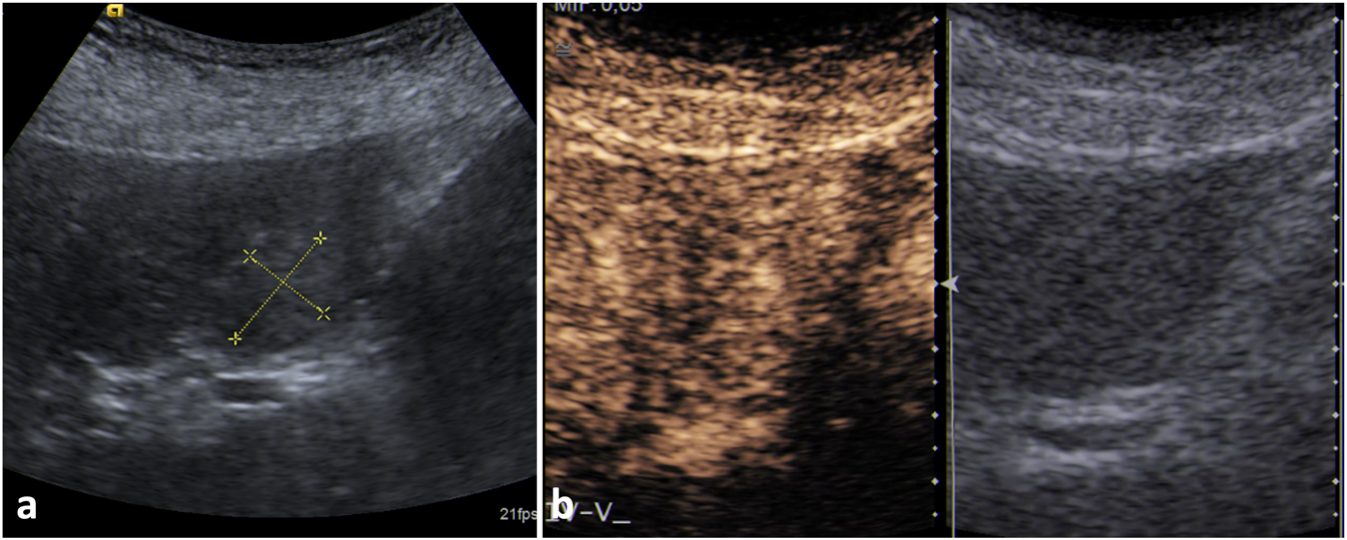
Focal steatosis. Focal steatosis appears mildly hyperechoic on B-mode US (**a**) and shows the same enhancement pattern of the adjacent liver in every phase on contrast-enhanced US (**b**)

Focal hyperechogenicity may be misinterpreted in the US as liver hemangioma (Fig. 5).

#### Tricks to avoid misinterpretation

The hyperechoic area has ill-defined margins, no mass-effect, and no acoustic enhancement. In case of doubts, CEUS may be used as a problem-solving technique demonstrating iso-enhancement.

### Inflammatory Hepatocellular Adenoma

#### Histopathologic features

Adenomas are rare benign hepatocellular tumors [37]. The inflammatory subtype (IHA) represents 30-50% of hepatocellular adenomas and is mainly seen in women [38–40]. Histologically, IHA is characterized pleomorphic hepatocytes proliferation with polymorphous inflammatory infiltrates, thickened tortuous arteries, and sinusoidal dilatation; consequently, they have a high bleeding risk. Prominent ductal reaction represents the distinct histological feature, whereas fat content is variable.

#### Typical imaging features

In the US, IHA mostly appears hyperechoic, surrounded by a hypoechoic halo [41]; CEUS shows a hypervascular pattern, similar to FNH. On unenhanced CT, IHAs show variable density, depending on the presence of hemorrhage [8]. On MRI, IHA usually appears iso/mildly hyperintense on T1-weighted images and heterogeneously hyperintense on T2-weighted images with a peripheral rim of high T2 signal intensity, the so-called “atoll sign” [42]. Mild signal drop on out-of-phase T1-weighted images may be present. In contrast-enhanced imaging studies, IHA shows hyperenhancement in the arterial phase followed by iso-intensity/density during the following phases of the dynamic study. On hepatobiliary phase MR images the lesion is typically hypointense, but iso/hyperintensity may be observed in about 20% of the cases [8].

HNF 1 alpha mutated liver adenomas are the second most common typically have a more prominent signal drop on out-of-phase T1-weighted images, appear mildly hyperintense on T2-weighted images and have a more moderate arterial enhancement with subsequent washout.

Beta catenin mutated, and unclassified liver adenomas are rare and do not have distinctive imaging features.

#### Misleading imaging features

**Fig. 6:**
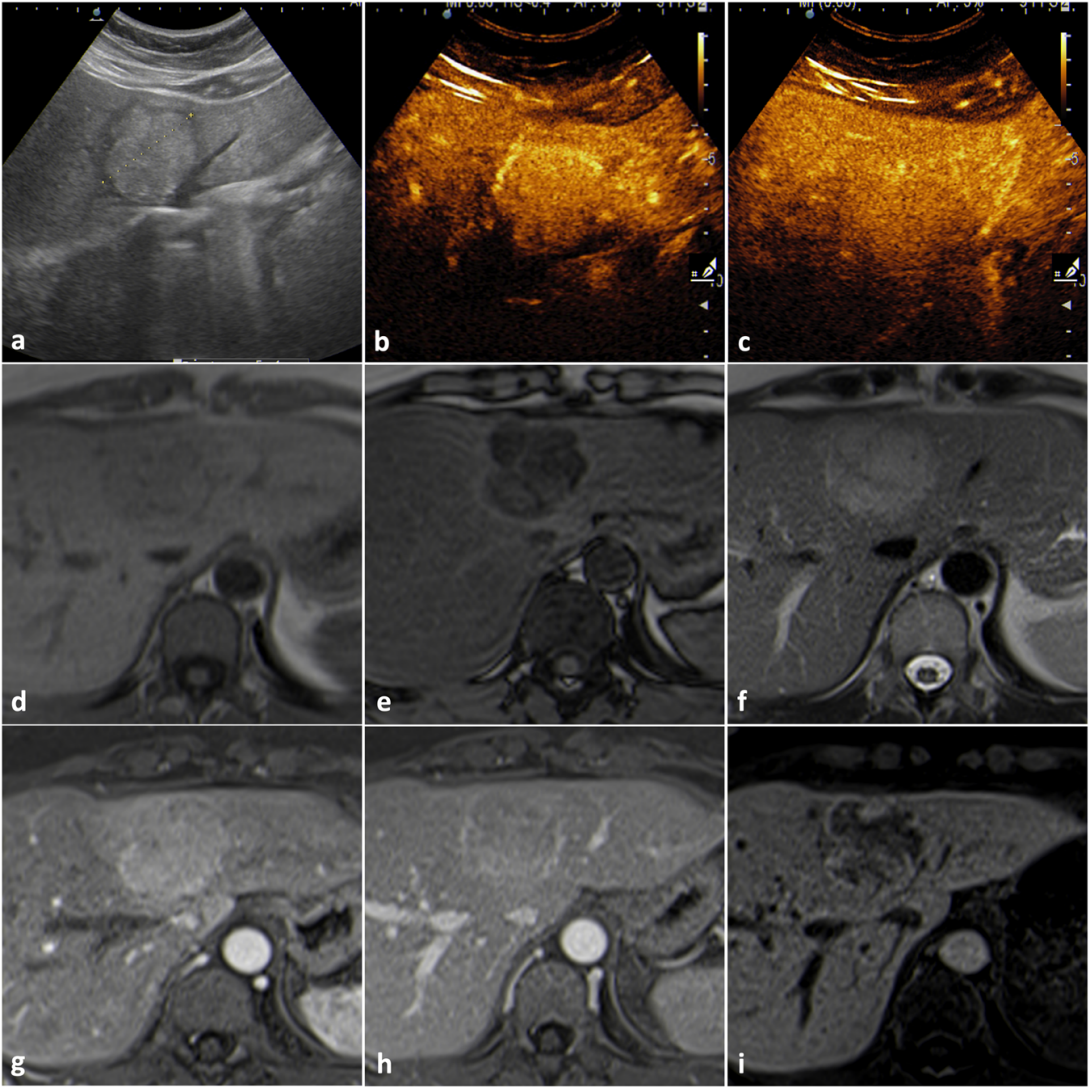
Inflammatory adenoma (biopsy-proven and resected). B-mode US (**a**) shows a lobulated hyperechoic mass surrounded by a hypoechoic halo. Contrast-enhanced US (**b**, **c**) shows homogeneous arterial enhancement without washout. On MRI, the lesion appears isointense on T1-weighted images (**d**) with a signal drop in the out-of-phase T1-weighted acquisition (**e**) and slightly hyperintense on T2-weighted images (**f**). The lesion shows homogeneous enhancement in the arterial phase (**g**), minimal washout in the portal venous phase (**h**) and appears hypointense in the hepatobiliary phase (**i**)

In the US, hyperechogenicity may be misinterpreted (Fig. 6), as well as the arterial hyperenhancement without washout on contrast-enhanced imaging studies; moreover, some inflammatory adenomas may appear markedly hyperintense on T2-weighted images. Differential diagnosis may be more difficult in the case of small lesions that may show enhancement patterns resembling capillary hemangiomas.

#### Tricks to avoid misinterpretation

In the US, the presence of a peripheral hypoechoic halo virtually excludes the diagnosis of hemangioma. Moreover, the arterial hyperenhancement on contrast enhanced imaging studies has no globular pattern, which must be present in every hemangioma > 2 cm.

### Arteriovenous fistula

#### Histopathologic features

An arteriovenous fistula (AVF) is an abnormal communication between the hepatic arterial and portal venous systems. The abnormal communication may be the consequence of different physiopathologic mechanisms that may involve the sinusoids, the vasa vasorum located between hepatic artery and portal vein branches, and the interlobular veins [43, 44].

#### Typical imaging features

Regardless of the AVF’s mechanism, the typical dynamic CT and MRI feature is the presence of nodular arterial hyperenhancement associated with early enhancement of peripheral portal vein branches, preceding main portal vein enhancement. US, CT, and MRI show no solid focal lesions.

#### Misleading imaging features

**Fig. 7:**
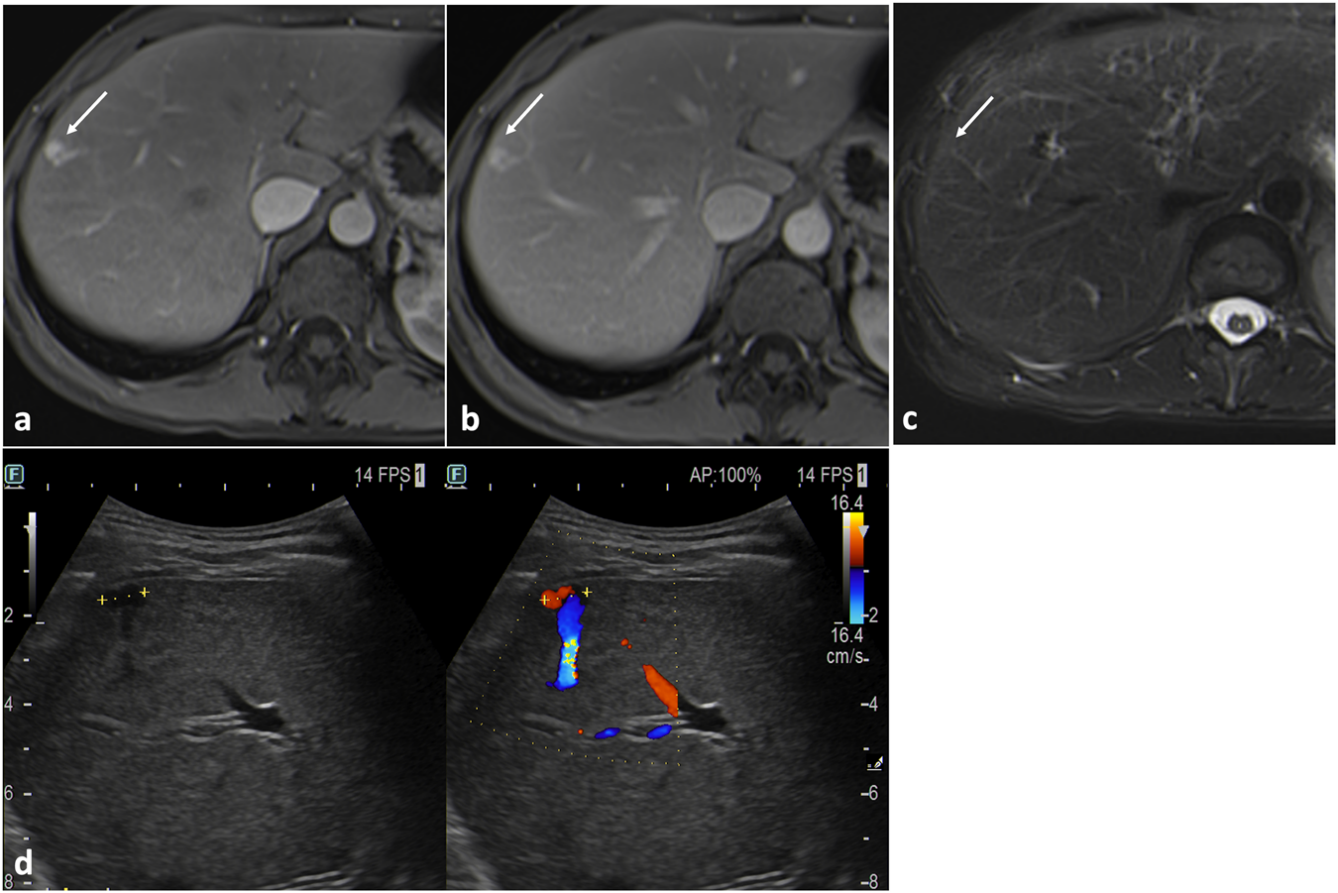
Artero-venous fistula. A focal hypervascular nodule (arrow) is appreciable on arterial phase T1-weighted images (**a**) between an arterial and a portal branch. The nodule shows the same signal intensity of adjacent vessels in portal venous phase (**b**), but no focal lesion can be delimited on fat-saturated T2-weighted images (**c**). On US (**d**) an anechoic round lesion with arterial flow signal on color-Doppler can be observed

Focal arterial hyperenhancement may be misinterpreted as capillary hemangioma (Fig. 7).

#### Tricks to avoid misinterpretation

No solid lesions are visible on pre-contrast MRI. Doppler US can be used to identify the fistula point.

### Angiomyolipoma

#### Histopathologic features

Angiomyolipoma (AML) is a rare benign tumor with marked female predilection [45]. Patients with multiple AMLs may be affected by tuberous sclerosis [46]. Histologically, AML is a capsulated mesenchymal tumor composed by smooth muscle cells, thick-walled blood vessels, and mature adipose tissue [47].

#### Typical imaging features

Given its heterogeneous macroscopic fat content, AMLs usually appear heterogeneously hyperechoic on the US, show areas of fat density on unenhanced CT, and appear markedly hyperintense on T1-weighted and T2-weighted images, with correspondent hypointensity after fat saturation. Contrast-enhanced studies show heterogeneous enhancement of the non-fatty components in the arterial phase [6, 47].

#### Misleading imaging features

**Fig. 8:**
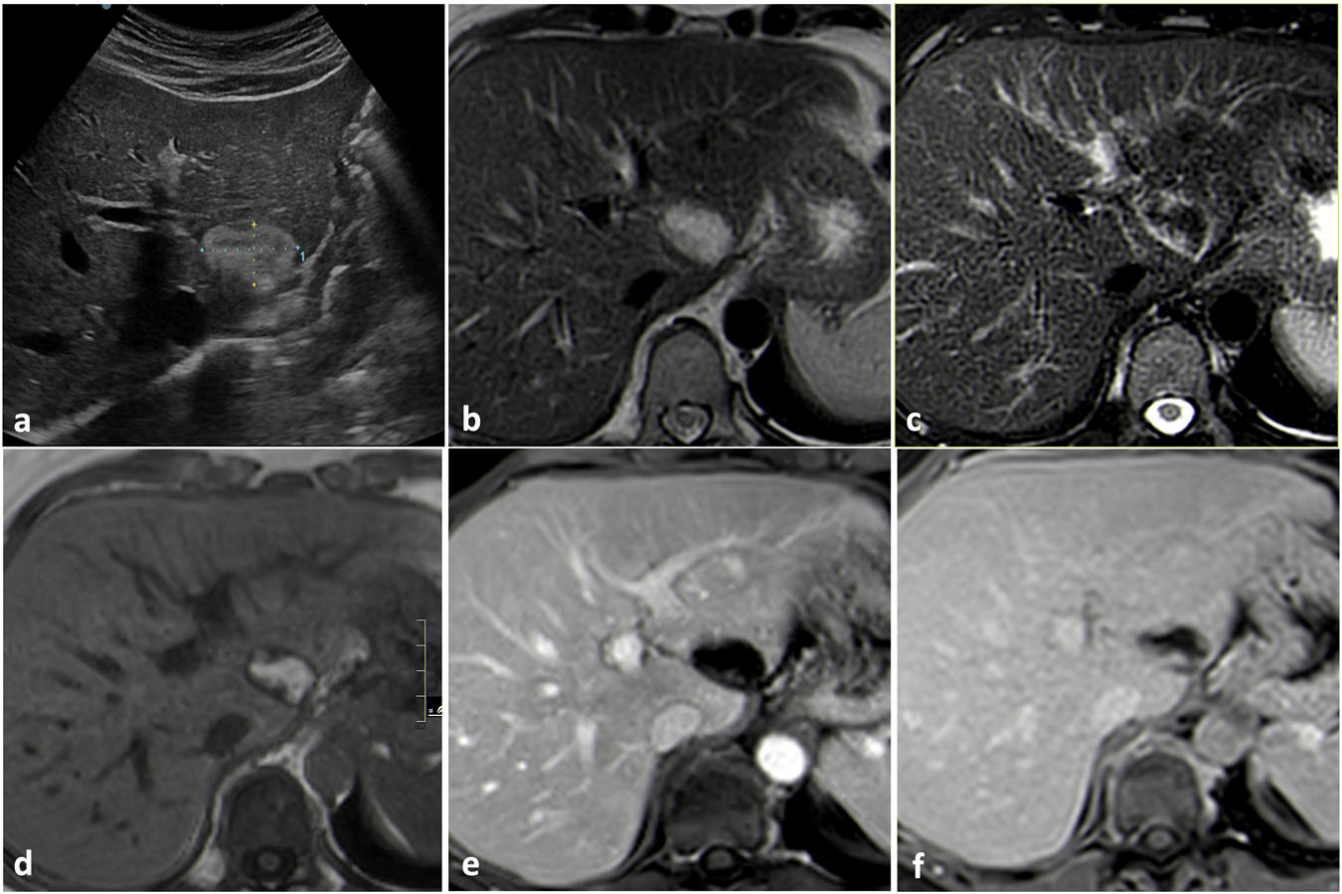
Angiomyolipoma. On B-mode US (**a**) a heterogeneous hyperechoic lesion is appreciable. The lesion appears markedly hyperintense on T2-weighted images (**b**) becoming prevalently hypointense after fat saturation (**c**) because of the macroscopic fatty content. On T1-weighted images (**d**) the lesion appears heterogeneously hyperintense; the peripheral components resemble a globular enhancement pattern during arterial (**e**) and portal venous (**f**) phases

Hyperechogenicity on the US, as well as marked T2-hyperintensity and peripheral enhancement on MRI may lead to misinterpretation as liver hemangioma (Fig. 8).

#### Tricks to avoid misinterpretation

Detection of discrete macroscopic fat components on CT or MRI is crucial to reaching the correct diagnosis; however, attention must be paid to the differential diagnosis with fat-containing malignant lesions (e.g., HCC).

### Peliosis

#### Histopathologic features

Peliosis hepatis is an extremely rare condition characterized by the presence of multiple blood-filled cysts in the liver parenchyma [48]. It is the consequence of hepatocellular necrosis that determines sinusoids’ wall damage and central vein dilatation [49].

#### Typical imaging features

Given the rarity of these lesions, variable imaging features have been described in different case reports. In the US, the vascular lakes appear hypo-/anechoic with possible flow signal on Doppler examination [50]. On unenhanced CT, the lesions have been mostly described as hypodense, whereas on MRI they usually appear hypointense on T1-weighted images and hyperintense on T2-weighted ones. After contrast material injection, progressive centrifugal enhancement has been mostly described, but the centripetal pattern may also be observed [48].

#### Misleading imaging features

Given the variable imaging appearance described in the literature, it is not possible to identify peculiar misleading features.

#### Tricks to avoid misinterpretation

Among the variable imaging features that have been described for peliosis, globular centripetal enhancement has been never observed; therefore, its absence in lesions larger than 2 cm enables to rule out hemangiomas.

## Malignant mimickers

### Intrahepatic cholangiocarcinoma

#### Histopathologic findings

Cholangiocarcinoma (CCC) is the second most common primary liver malignancy after hepatocellular carcinoma, and “mass-forming” represents the most common growth pattern [51–53]. Intrahepatic CCC arises from the epithelium lining second-order bile ducts. Histologically, tumor cells are in the periphery of the tumor, whereas a dense fibrous stroma occupies the central portion.

#### Typical imaging findings

Mass-forming CCC usually appears as a large lobulated mass with irregular margins and may determine capsular retraction. In the US, mass-forming CCC typically shows intermediate echogenicity surrounded by a hypoechoic halo [54], whereas on unenhanced CT it appears hypodense in comparison to the adjacent liver. Given its histologic composition, on MRI, the lesion usually shows a T2-hyperintense peripheric rim with increased diffusion restriction associated to a T2-hypointense core with no increased diffusion restriction. On contrast-enhanced CT and MRI, mass-forming CCC shows a peripheral rim enhancement in the arterial phase, which in fact is completely different of the globular pattern observed in hemangiomas, followed by gradual irregular centripetal enhancement during the following phases of the dynamic study [53, 55], whereas on CEUS, given the pure intravascular distribution of the contrast material, arterial peripheral enhancement is not followed by centripetal filling of the fibrotic tissue [56]. During hepatobiliary phase MRI, the fibrotic tissue may retain contrast given its large extracellular spaces [57].

#### Misleading imaging features

**Fig. 9:**
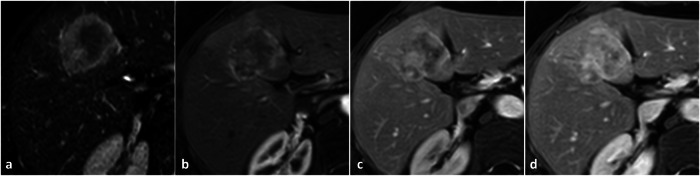
Intrahepatic cholangiocarcinoma (biopsy-proven and resected). The lesion shows heterogeneous signal intensity on T2-weighted images (**a**) with peripheral hyperintensity associated with central hypointensity. During the dynamic study, peripheral rim enhancement is appreciable in the arterial phase (**b**), followed by centripetal filling in the portal venous (**c**) and equilibrium (**d**) phase

The presence of peripheral enhancement followed by centripetal filling on CT and MRI may be misinterpreted as liver hemangioma (Fig. 9).

#### Tricks to avoid misinterpretation

Although being centripetal, the enhancement pattern is not globular and is more ring-like. The lesion appears mainly hypointense on T2-weighted images. Increased diffusion restriction should not be present in hemangiomas.

### Mucinous colorectal metastases

#### Histopathologic features

The mucinous variant represents 5–15% of colorectal cancers [58] and is histologically characterized by the presence of at least 50% of extracellular mucin pools [59, 60].

#### Typical imaging features

Imaging features of mucinous colorectal liver metastases are the direct consequence of their high mucin content. Mucinous colorectal metastases typically appear hyperechoic on the US [61], markedly hypodense on unenhanced CT, and markedly hyperintense on T2-weighted images with shine-through artefact con DWI and hyperintensity on the ADC map. On CT and MRI, peripheral rim enhancement in the arterial phase followed by centripetal filling is usually recognizable, whereas on CEUS peripheral rim enhancement is not followed by centripetal filling. The mucinous material, given its large extracellular spaces, may retain contrast on hepatobiliary phase MRI.

#### Misleading imaging features

**Fig. 10:**
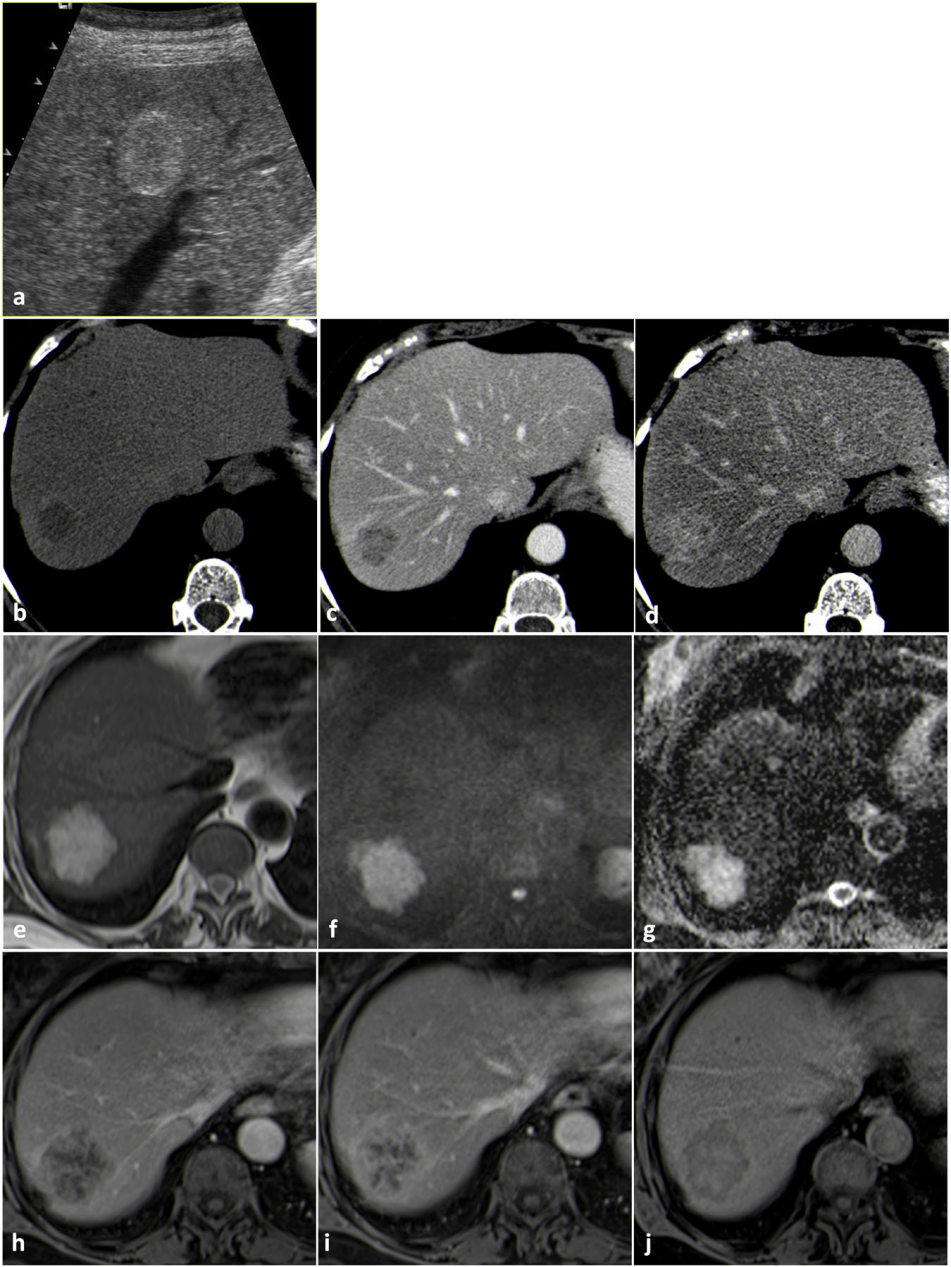
Mucinous colorectal cancer metastasis (biopsy-proven and resected). The lesion appears heterogeneously hyperechoic on B-mode US (**a**). On CT the lesion appears markedly hypodense in the native phase (**b**) and shows progressive enhancement in the portal venous (**c**) and equilibrium (**d**) phase. On MRI the lesion appears markedly hyperintense on T2-weighted images (**e**), determining shine through artifact on high b-value DWI (**f**) without true diffusion coefficient restriction on ADC map (**g**). The lesion shows peripheral enhancement in late arterial phase (**h**) with progressive centripetal filling in portal venous phase (**i**). In hepatobiliary phase (**j**) the central component shows contrast material retention

Hyperechogenicity in the US, marked T2-weighted hyperintensity on MRI, and peripheral arterial enhancement with centripetal filling, both on CT and MRI, may lead to misdiagnosis (Fig. 10).

#### Tricks to avoid misinterpretation

Peripheral arterial enhancement without globular pattern enables to exclude the possibility of a hemangioma.

### Angiosarcoma

#### Histopathologic features

Angiosarcoma is a rare mesenchymal hepatic malignant tumor, accounting for less than 2% of all primary liver neoplasms [62]. Histologically, it consists of fusiform or pleomorphic cells developing in pre-existing vascular spaces. Angiosarcoma is often multifocal at the time of the diagnosis and extra-hepatic localizations, typically within the spleen and lungs, are usually present.

#### Typical imaging features

In the US, angiosarcoma appears heterogeneously hyperechoic, and intralesional cystic spaces are usually present. Angiosarcoma appears heterogeneously hypodense on CT and heterogeneously hyperintense on T2-weighted images with a heterogeneous increase of diffusion restriction [63]. The lesions show irregularly shaped intralesional or peripheral foci of enhancement during the arterial phase, with progressive incomplete enhancement on portal venous and delayed phases, both on CT and MRI.

#### Misleading imaging features

**Fig. 11:**
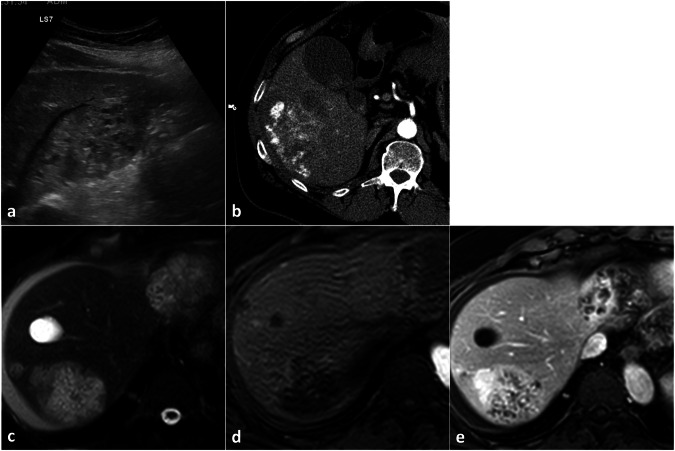
Angiosarcoma (biopsy-proven). B-mode US (**a**) shows an ill-defined hyperechoic lesion with multiple cystic lacunae. Multiple discrete foci of increased enhancement are appreciable on arterial phase CT (**b**). On T2-weighted images (**c**) two large heterogeneously hyperintense lesions and at least to homogeneously hyperintense satellite nodules are appreciable. The lesions show peripheral foci of increased enhancement in the arterial phase (**d**) followed by centripetal filling with multiple avascular lacune in the portal venous phase (**e**)

The foci of arterial enhancement might in some way simulate liver hemangiomas’ enhancement globules both on CT and MRI (Fig. 11).

#### Tricks to avoid misinterpretation

The arterial enhancement foci lose their eventual globular shape during the following phases of the dynamic studies. Irregular avascular areas and perilesional satellite nodules are usually present. T2-hyperintensity is mild and heterogeneous.

### Hemangioendothelioma

#### Histopathologic features

Hemangioendothelioma is a rare, malignant sarcomatous tumor of endothelial origin [64]. Histologically, the vacuolated endothelial cells are arranged in short cords and strands in a background of myxohyaline stroma [65]. Multiple lesions are usually present at the time of diagnosis.

#### Typical imaging features

The lesions usually show heterogeneous density on CT and heterogeneous signal intensity on MRI [66–68]. On contrast-enhanced imaging, various patterns have been described [69]: (a) peripheral nodular arterial enhancement followed by wash-out, (b) rim-like arterial enhancement with wash-out during the portal venous phase, and (c) inversed target sign with/without wash-out during the portal venous phase.

#### Misleading imaging features

**Fig. 12:**
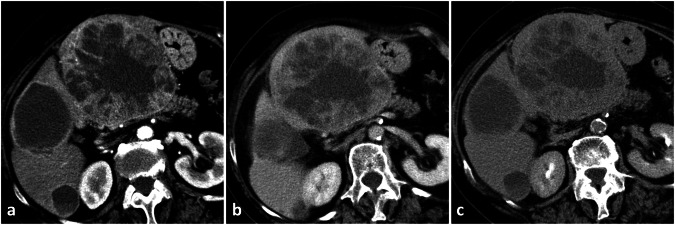
Hemangioendothelioma (biopsy-proven). Three markedly hypodense lesions with moderate peripheral enhancement are appreciable in late arterial phase CT (**a**); mild progressive centripetal filling is recognizable in portal venous (**b**) and delayed (**c**) phase, with persisting large hypodense areas

Enhancement pattern during the arterial phase may be misinterpreted (Fig. 12).

#### Tricks to avoid misinterpretation

Large avascular lacunae are usually present. The enhancement pattern is not globular.

## Conclusions

Liver hemangiomas have many benign and malignant mimickers showing one or more imaging features resembling hemangiomas, but all of them also show imaging features that enable to avoid misinterpretation. The main difficulties in the differential diagnosis between liver hemangiomas and their benign mimickers arise on US examination; in these cases, contrast material administration is fundamental to avoid pitfalls. On the other hand, malignant mimickers mostly show misleading features on contrast-enhanced CT and MRI; in these cases, comprehensive “multiparametric” evaluation of the available imaging features is crucial to achieve a correct diagnosis.

## Supplementary information

ELECTRONIC SUPPLEMENTARY MATERIAL

## Data Availability

All radiological images are available in the Institutional PACS.

## Abbreviations

AML: Angiomyolipoma
AVF: Arteriovenous fistula
CCC: Cholangiocarcinoma
FNH: Focal nodular hyperplasia
IHA: Inflammatory adenoma
