# Supplementary material for: I thought it was a hemangioma! A pictorial essay about common and uncommon liver hemangiomas’ mimickers
Source: Insights Imaging. 2024 Sep 19;15:228. doi: 10.1186/s13244-024-01745-1 (PMC11413281; doi:10.1186/s13244-024-01745-1)
Supplement: Supplementary file 1 — ELECTRONIC SUPPLEMENTARY MATERIAL [file 13244_2024_1745_MOESM1_ESM.pdf]

I thought it was a hemangioma! A pictorial essay about common and uncommon liver hemangiomas' mimickers.

ELECTRONIC SUPPLEMENTARY MATERIAL

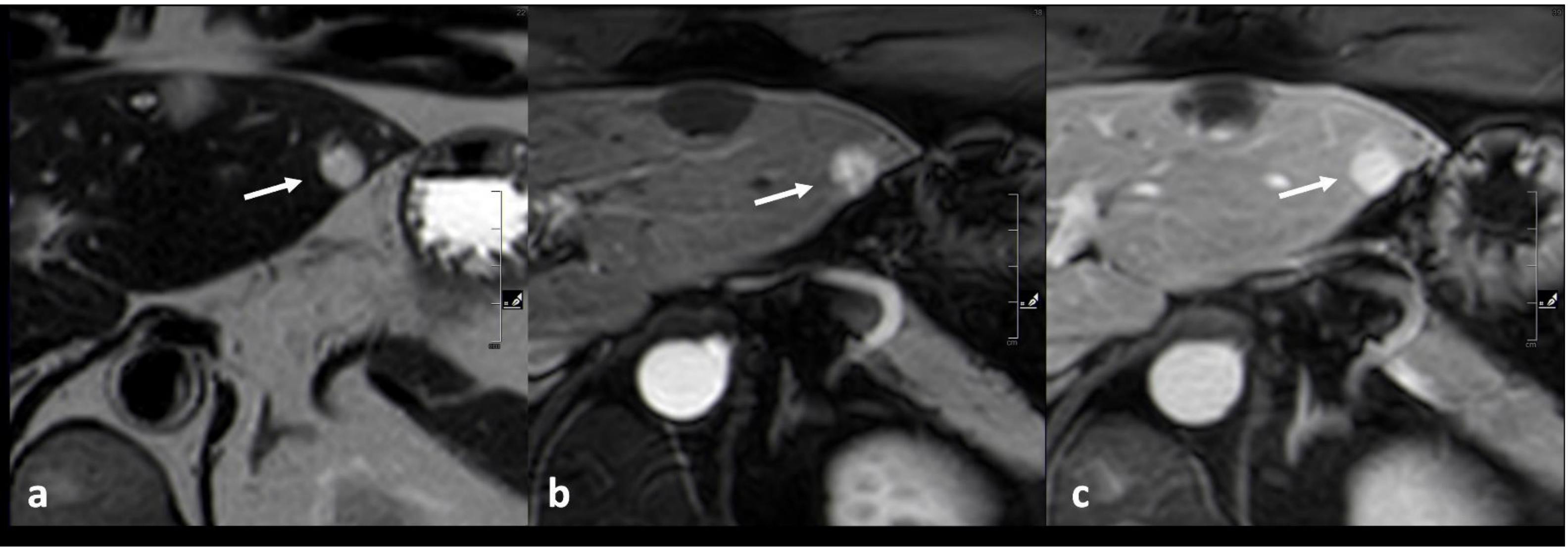

Fig.S1 Typical MRI findings in capillary hemangioma. The small lesion (arrows) appears markedly hyperintense on T2-weighted images (a), shows intense arterial hyper-enhancement (b) and appears isointense to the adjacent vessels in portal venous phase acquisition (c).

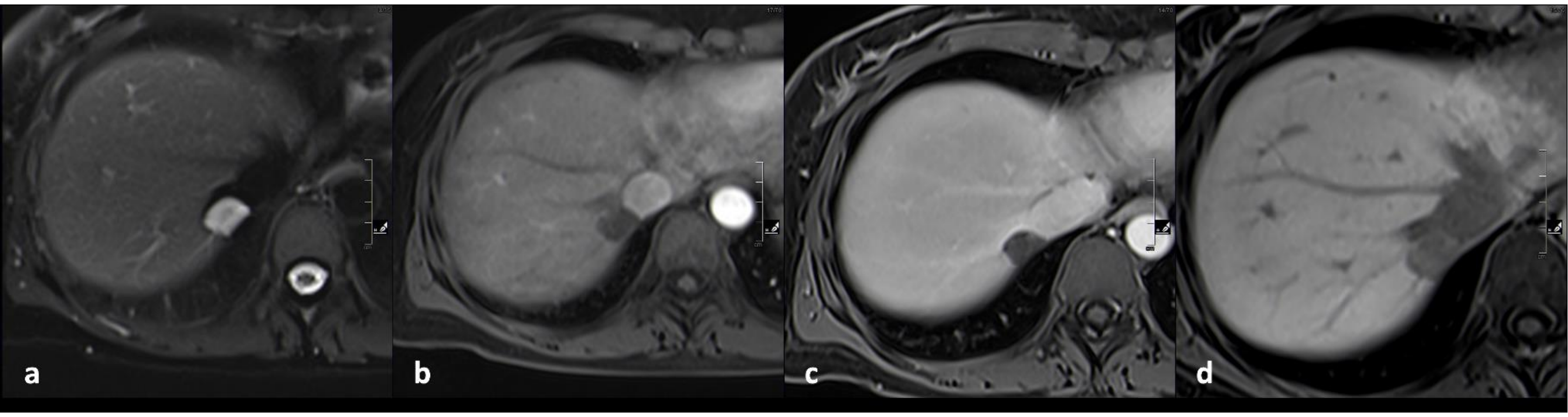

Fig.S2 MRI findings in sclerosed hemangioma (biopsy-proven). The lesion appears prevalently markedly hyperintense on T2-weighted images (a) with a relatively hypointense core. After contrast material injection the lesion shows no enhancement in arterial (b) and equilibrium phases (c). The lesion appears hypointense in comparison to adjacent liver in hepatobiliary phase (d).
